# Capturing Free Surface Dynamics of Flows over a Stepped Spillway Using a Depth Camera

**DOI:** 10.3390/s25082525

**Published:** 2025-04-17

**Authors:** Megh Raj K C, Brian M. Crookston, Daniel B. Bung

**Affiliations:** 1 Utah Water Research Laboratory, Department of Civil and Environmental Engineering, Utah State University, 8200 Old Main Hill, Logan, UT 84322-8200, USA; megh.kc@usu.edu; 2 Hydraulic Engineering Section, Civil Engineering Department, FH Aachen University of Applied Sciences, 52066 Aachen, Germany; bung@fh-aachen.de

**Keywords:** RGB-D camera, stereoscopic vision, depth measurement, stepped spillway, surface fluctuations

## Abstract

Spatio-temporal measurements of turbulent free surface flows remain challenging with in situ point methods. This study explores the application of an inexpensive depth-sensing RGB-D camera, the Intel^®^ RealSense™ D455, to capture detailed water surface measurements of a highly turbulent, self-aerated flow in the case of a stepped spillway. Ambient lighting conditions and various sensor settings, including configurations and parameters affecting data capture and quality, were assessed. A free surface profile was extracted from the 3D measurements and compared against phase detection conductivity probe (PDCP) and ultrasonic sensor (USS) measurements. Measurements in the non-aerated region were influenced by water transparency and a lack of detectable surface features, with flow depths consistently smaller than USS measurements (up to 32.5% less). Measurements in the clear water region also resulted in a “no data” region with holes in the depth map due to shiny reflections. In the aerated flow region, the camera effectively detected the dynamic water surface, with mean surface profiles close to characteristic depths measured with PDCP and within one standard deviation of the mean USS flow depths. The flow depths were within 10% of the USS depths and corresponded to depths with 80–90% air concentration levels obtained with the PDCP. Additionally, the depth camera successfully captured temporal fluctuations, allowing for the calculation of time-averaged entrapped air concentration profiles and dimensionless interface frequency distributions. This facilitated a direct comparison with PDCP and USS sensors, demonstrating that this camera sensor is a practical and cost-effective option for detecting free surfaces of high velocity, aerated, and dynamic flows in a stepped chute.

## 1. Introduction

Turbulent free-surface flows through hydraulic structures, such as those observed in spillway chutes and stilling basins, are a significant measurement challenge due to intricate flow geometries, surface roughness, fluctuating water surfaces, and splash and spray. These flows are often characterized by intense mixing and water surface deformation leading to surface breakup, self-aeration, and a complex multiphase mixture of air and water [1,2,3] with highly transient interfacial boundaries. The need to characterize the spatio-temporal water surface and relevant air–water flow properties has led to the development and application of a variety of instruments and sensors primarily used in scaled physical models and test facilities in the laboratory [4].

Classical devices for water surface measurements, such as wire gauges [5,6] and point/staff gauges [7,8,9], can be subjective [1]. Intrusive phase detection probes are well-established for internal air–water features and come in multiple versions, such as optical [10,11] and conductivity probes [12,13,14,15], often requiring sophisticated instrumentation and analyses where the water surface is estimated to be where water is detected only 10% in a time series. As noted by Kramer and Bung [16], remote sensing methods present a new frontier to complement established devices or improve the characterization of these complex turbulent free-surface flows. Nonintrusive sensors such as acoustic displacement sensors (e.g., ultrasonic sensors) [17,18] and laser-based systems (e.g., various forms of Light Detection and Ranging (LiDAR)) [16,19] provide new capabilities for analyzing these flows. Furthermore, optical techniques, such as photogrammetry [20,21] and RGB imaging [22], high-speed cameras [22,23,24], particle tracking [25], and various classes of LiDAR [26], enable the scanning of large, complex water surfaces. Table 1 presents a selection of key studies leveraging non-intrusive methods to investigate turbulent free-surface flows in hydraulic structures. Certainly, the collective literature highlights the diversity of approaches used in surface flow measurements, yet it remains a relatively new frontier both in usage and in the characterization of what a device is capturing and what is represented in the data [16].

Each of the aforementioned techniques offers specific advantages and limitations depending on the application and the flow conditions, including costs associated with acquiring and using expensive scientific equipment. Bung et al. [31] applied, for the first time, the Intel^®^ RealSense™ D435 RGB-D camera sensor to the classical hydraulic jump case, demonstrating the viability of an inexpensive RGB-D sensor for measuring turbulent free-surface flows. Nonetheless, their results only included the center transect or a two-dimensional slice for a finite range of flows. More recently, Steinke and Bung [25] applied the RGB-D camera with other sensors to study the water surface of an undular hydraulic jump. Both studies highlighted the need for appropriate texture at the surface to be recognized. While such texture may also be obtained by dye or surface tracers, the studies focused on aerated flows with highly turbulent, splashing surfaces. It is acknowledged that this air–water interaction at the free surface implicates significant disturbances and, thus, increases uncertainty. However, it could be demonstrated that this uncertainty is in the same order as for classical air–water flow instrumentation [31]. It is stressed that, by now, only stereo vision cameras can provide instantaneous 3D data, which is a significant improvement to commonly employed sensors (e.g., ultrasonic sensors) or even LiDAR. The authors are unaware of any other literature where an RGB-D camera was used to study water surfaces in hydraulic structures; therefore, to build upon these two recent advancements, this study considered the performance of the Intel^®^ RealSense™ D455 RGB-D camera [32] to investigate, in three-dimensions, flow over a stepped spillway, leveraging its ability to capture detailed surface measurements across a broad field. This is a challenging case as flows in a stepped spillway include three distinct regions: (1) an unaerated clear water region [9,33], (2) an inception point [34,35] or transition region that includes self-aeration with splash and spray [36], and (3) an aerated white-water region [13,37] with an undistinguished irregular free surface with much splash and spray.

This study also included an assessment of appropriate ambient lighting conditions and explored various sensor settings and parameters to describe the capabilities of the Intel^®^ RealSense™ D455 RGB-D camera for spatio-temporal measurements in the stepped spillway. RGB-D free-surface measurements were compared directly to two additional datasets collected in this study via an ultrasonic sensor (USS) and a phase-detection conductivity probe (PDCP). These results provided further insights into what this RGB-D sensor is detecting, its capabilities and limitations, and if it may complement these other established sensors for studying the water surface of complex air–water flows.

## 2. Materials and Methods

### 2.1. Experimental Set-Up

**Figure 1:**
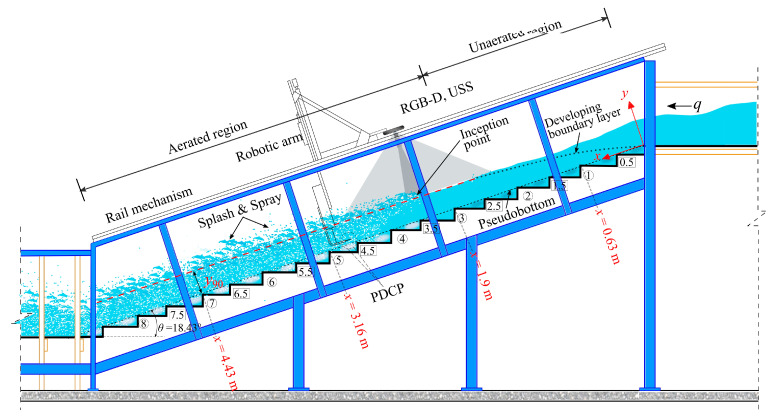
Side-view of the experimental setup illustrating the positioning of the RGB-D camera and USS above the water surface. In situ measurement locations with the PDCP, step edge identification, and select streamwise distances also shown.

Experiments were conducted at the Utah Water Research Laboratory (UWRL) at Utah State University (USU) utilizing a 1 m wide (*W*) stepped spillway with a slope angle (*θ*) of 18.43°. The spillway featured square-edge steps of uniform step height, *h* = 0.1 m (Figure 1). Discharge (*Q*) was supplied to the spillway via a headbox fitted with a flow straightener and a diffuser pipe, and *Q* was accurately measured using a Venturi flow meter (installed in the supply pipe connecting the headbox) and a pressure transducer with a measurement accuracy of ±0.25%.

A unit discharge *q* = 0.285 m^3^/(sm) was selected as it well-positioned the inception point within the flume, as illustrated in Figure 1. This *q* corresponds to a dimensionless ratio *d_c_*/*h* = 2.0 [where *d_c_* = (*q*^2^/*g*)^1/3^ is the critical flow depth with *g* denoting the gravitational acceleration] and a Reynolds number Re = 0.97 × 10^−6^ ($Re=V_{m}\times D_{h}/\upsilon$ where $V_{m}=q/y_{cw}$ and $D_{h}$ is the hydraulic diameter and *υ* is the kinematic viscosity of water and $y_{cw}$ is the corresponding clear water flow depth).

**Figure 2:**
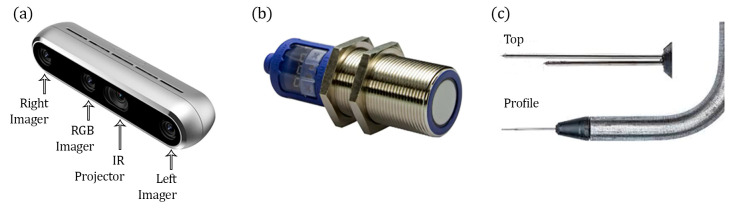
Sensors used in this study are (**a**) the Intel^®^ RealSense^TM^ Depth Camera D455 (RGB-D), (**b**) the Microsonic mic + 130 ultrasonic sensor (USS), and (**c**) the dual-tip phase detection conductivity probe (PDCP) tips.

This flow condition was investigated using three different measurement techniques to characterize the free-surface and air–water flow properties: (1) an Intel^®^ RealSense™ D455 RGB-D camera (Microsoft, Santa Clara, CA, USA), (2) a Microsonic mic+ 130 ultrasonic sensor (USS) (Microsonic, Dortmund, Germany), and (3) a dual-tip phase detection conductivity probe (PDCP) (Sydney, Australia) (see Figure 2). For reference, visual markings of the water surface were made on the spillway viewing window (left sidewall). The PDCP was oriented to the angle of the spillway in a streamwise direction (*x*) and was equipped with two identical needle tips of diameter *Ø* = 0.6 mm with inner electrodes of *Ø* = 0.125 mm; tip separation was precisely 5.24 mm. The air concentration *C* measurements with the PDCP were based on the leading tip signal. The PDCP operates by utilizing the difference in electrical conductivity between air and water. As the air–water flow passes over a probe tip, air bubbles occasionally contact the tip. Each time a bubble makes contact, the probe’s electrical signal changes, resulting in corresponding variations in the electrical signals between the air and water phases. The time series of this signal is then processed to calculate air–water flow properties, including air concentration and velocity, and air bubble characteristics [38,39].

A rail mechanism installed on top of the spillway sidewalls facilitated the longitudinal movement of a robotic arm (CNC controller and computer) equipped with measuring devices (RGB-D camera, USS, and PDCP). The devices were precisely positioned and verified; an automated scheme was formulated for the movement of the robotic arm in the streamwise (*x*) and vertical (*y*) axes with the USS and the PDCP tips corresponding to each step edge.

Measurements from each sensor were conducted independently due to constraints that prevented the simultaneous placement of all instruments at the same measurement location. For the optical measurements, the Intel^®^ RealSense™ D455 RGB-D camera was positioned at a distance of 0.98 m from the pseudobottom (see Figure 1) with a field-of-view (FoV) that captured aerated and unaerated portions of the flow along with the inception point of aeration. The plane of the camera was aligned parallel to the pseudobottom along the streamwise direction to an accuracy of <0.13° with a digital level and corroborated with sensor measurements in dry conditions. The camera was connected to the working laptop using a high-speed 3 m long USB C-to-A cable, but data acquisition was slightly improved with shorter cables that reduced periodic random frame loss. Further details on the camera’s working principle, settings, sensitivities, internal calibration procedures, etc., are detailed in Section 2.2, Section 2.3 and Section 2.4 herein.

Similarly, along the spillway centerline, a single USS equipped with a shield cone (to protect the membrane from splash and spray) was placed sequentially above step edges at a distance of 0.96 m. USS water surface measurements were made at a sampling frequency of *f =* 200 Hz for a sample duration of *T* = 180 s per location [12]. The transducer frequency of the ultrasonic sensor was 200 kHz, the measuring range was 0.2–2.0 m, the accuracy was ±1%, and the response time was about 92 ms. Although the USS is commonly regarded as a point-based measuring device, it emits an ultrasonic pulse in a conical beam. As the pulse travels outward, the beam diameter increases with distance from the sensor, effectively creating a coverage angle or detection cone. This means the sensor may receive reflections from multiple points within its footprint, particularly if it is in the context of a turbulent water surface or ejected water droplets. Indeed, the USS reading can be influenced by surface deformations, splashes, or lateral flow motions present within this detection zone.

Finally, intrusive measurements with the PDCP were also collected along the spillway centerline. Vertical profiles (5 mm intervals above the pseudobottom) at each step edge were sampled for *T* = 90 s at *f =* 200 kHz based on a sensitivity analysis of different parameters (velocities, air concentrations, and turbulence). The PDCP measurements required a relatively larger sampling frequency than those of the USS due to the PDCP’s operational principle, which relies on the detection of rapidly fluctuating air–water interfaces in the water column.

### 2.2. Intel^®^ RealSense^TM^ RGB-D Camera Overview and Working Principle

Intel^®^ RealSense^TM^ cameras, such as the D415, D435, and D455 [40], are relatively inexpensive depth sensors for 3D vision. PrimeSense originally developed the depth cameras in 2009 for use in video games [41], and, since then, this technology has found applications in several fields, such as face recognition, robotic navigation, toys, smart vehicles, etc. [42,43,44,45]. As noted earlier, the sensor used in this study was the Intel^®^ RealSense^TM^ RGB-D455 camera from the D400 series. It was released in 2020 and intended for high-precision depth measurement using stereoscopic vision. It supports both passive and active stereo modes, where the passive mode relies on sufficient ambient light to generate depth information, and the active mode emits its own infrared light (IR projector) in a random dot pattern for enhanced depth sensing in low-light or texture-poor environments. The D455 has a greater range over the D435 camera by a factor of two (Intel^®^ RealSense^TM^ Depth Camera D455, 2021), greater RGB FoV, and improved accuracy for similar distances. The use of global shutter sensors allows the camera to capture distortion-free images in highly dynamic scenes and reports to be capable of capturing fast-moving objects. The optical module is comprised of an RGB imager, an infrared projector, a left imager, and a right imager, as shown in Figure 2a. Additional main features of the D455 camera are summarized in Table 2.

**Figure 3:**
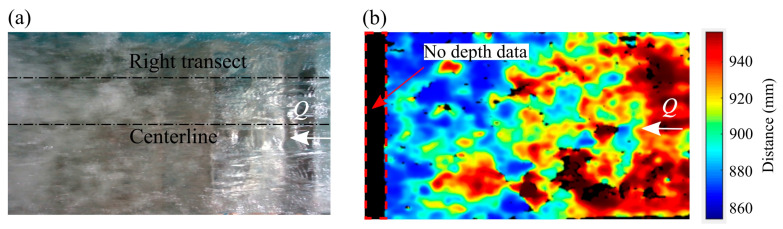
Intel^®^ RealSense^TM^ D455 RGB-D recording of the instantaneous water surface. (**a**) RGB image of the FoV and (**b**) corresponding depth map. Note that the depth map represents the measured distance of the water surface from the camera plane.

The camera assembly is equipped with an Intel^®^ RealSense™ Vision Processor D4, an advanced Application Specific Integrated Circuit (ASIC) that computes depth or distance [32]. The Vision Processor is based on 28 nm process technology to process information from the optical modules in real-time and to reduce latency without relying on external processing resources. The depth estimation by the camera is based on the principle of stereoscopic vision in which depth is calculated by comparing the simultaneously recorded images with distinguishable features from the left and right imagers, separated by a baseline distance of 95 mm. Each camera first captures pixelwise 2D images of their respective FoV. The system aligns these images along rectified epipolar lines on a shared virtual plane, enabling accurate comparison. Depth is then calculated by determining the disparity, which is the number of pixel shifts required to align features in the right image with those in the left. By triangulating these disparities, the system derives depth values for each pixel across the entire FoV. The stereo-matching algorithm uses the left imager as a reference, causing any non-overlapping areas on the left to produce a consistent no-data region in the resulting depth map, as seen in Figure 3. The pixel-wise depth estimation is calculated based on Equations (1) and (2) as follows:

(1) $$Depth=\frac{focal\;length\times baseline\;distance}{disparrity}$$

(2) $$focal\;length=\frac{X_{res}}{2\;tan\left(H_{FoV}/2\right)}$$

where $X_{res}$ is the horizontal image resolution in pixels and $H_{FoV}$ is the horizontal field of view. Due to their stereoscopic vision-based principle, depth estimation relies predominantly on the detection of distinct features by the camera for comparison, thereby meriting well-lit environments for accuracy and better performance.

### 2.3. Camera Settings

The Intel^®^ RealSense™ D455 has a cross-platform open-source software development kit (SDK) known as LibRealSense [46]. This SDK provides a variety of tools and functionalities to interact with the camera and customize its performance to meet specific application requirements. One of the main tools included in LibRealSense is the RealSense Viewer, a precompiled graphical interface that allows users to access and modify camera parameters and perform a system calibration. It provides live visualization of the depth and RGB streams, allowing immediate feedback to users when changing settings or confirming spatial positioning. Parameters that can be adjusted include but are not limited to camera resolution, frame rate, exposure, gain, laser power (i.e., the IR Projector, see Figure 2a), enabling or disabling the projector and cameras, and post-processing filters.

**Figure 4:**
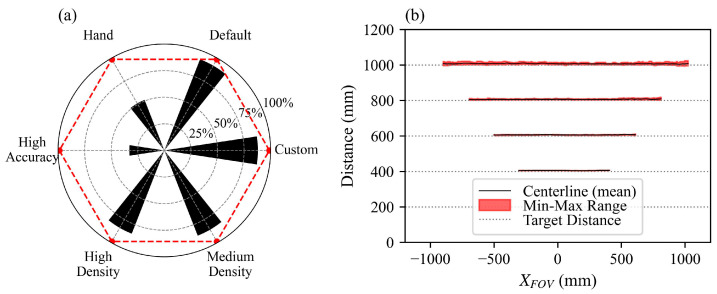
(**a**) Fill rate and measurement accuracy in percentage for different camera presets; red dots represent the measurement accuracy for a dry wall target and black radial bars show the fraction of FoV filled with depth data; (**b**) mean centerline measurements for a dry wall test at different target distances with a minimum and maximum range of readings considering a 10 s sample duration.

Our study involved a detailed evaluation of the camera’s six primary visual presets (custom, default, hand, high accuracy, high density, and medium density), each reportedly optimized for distinct operational scenarios or object detection schemes. A comparative analysis of these presets was performed herein based on the fill rate (the water surface) and measurement accuracy for a plane condition (a vertical wall). Default, custom, high-density, and medium-density presets provided a better fill rate, while the high-accuracy and hand presets generated a poor fill rate, as shown in Figure 4a. The results were obvious as the hand preset is designed for hand tracking and gesture recognition, whereas the high accuracy compromised the fill factor for better measurement accuracy. Based on iterative testing and optimization, we selected a custom preset for the best balance of accuracy and fill factor. While the manufacturer specifies a measurement error of 2% for distances up to 6 m, our observations for the plane condition indicated that measurement errors (at a distance from 0.8 m to 1 m) were well within 2% for all the presets with a 100% fill rate. The uncertainty in measured distances increased with increasing target distance (Figure 4b). This also highlights the sensitivity of this technique in dynamic environments, and the careful selection of camera presets and parameters cannot be ignored. For the challenging application of turbulent water surface measurements, we prioritized a high *f* to capture rapid surface fluctuations while maintaining a resolution of 848 × 480 pixels, with *f =* 90 Hz and *T* = 60 s. This configuration achieved the necessary FoV and spatial resolution to reliably capture water surface dynamics in our experimental setting. Additional parameter adjustments included setting the laser power to 250 mW, which is consistent with Bung et al. [31] and Carfagni et al. [41], and using a depth unit of 1 mm, a depth exposure of 33,000 μs, and a minimum image gain of 16. For further information on camera parameter selections, please see a summary in Table 3 or refer to Grunnet-Jepsen et al. [47] for more details.

In tandem with optimizing camera parameters, various light sources were considered to ensure effective illumination in the FoV. The resulting intensity of light due to the adopted light sources (see Table 4), in addition to standard laboratory fluorescent lights, was measured using a handheld URCERI MT-912 digital light meter (measuring range: 0–200,000 lux). The light sources resulted in at least 600 lux or greater intensity within the FoV of the camera. Placing an additional light source underneath the flume created illumination effects on the water surface in the non-aerated region that increased no-data points. Consequently, under-flume lighting was excluded during final measurements.

### 2.4. RGB-D Data Acquisition and Post-Processing

The Intel^®^ RealSense™ camera is available in a pre-calibrated, ready-to-use, configuration. Nonetheless, to test measurement precision and camera performance, a static wall test and a self-calibration were carried out as described in Grunnet-Jepsen et al. [48]. The RGB-D camera was placed to measure target distances of 400 mm, 600 mm, 800 mm, and 1000 mm with each measurement taken for *T* = 10s. Figure 4b shows the measurements along the centerline of the FoV. It was noticed that the measurement accuracy decreased with increasing target distance, with a measurement error of ≈0.7% for a target distance of 1000 mm. No noticeable distortion was evident along the centerline of the flat wall (depth values remained nearly constant), although the maximum and minimum ranges were slightly larger toward the edges of the FoV (minimal lens effect). Subsequently, the self-calibrated camera was installed in the experimental flume, precisely aligned with the centerline and parallel to the pseudobottom. Under dry conditions, a reference steel plate (~2.5 m long) of known thickness was placed in situ to verify the camera’s depth measurements in the FoV and establish the reference distance from the camera to the pseudobottom, which was independently measured. Upon confirming baseline accuracy, a series of wet tests were conducted with the same camera position to measure the water surface. The RGB-D recordings are saved as “.bag” files that were post processed and analyzed using MATLAB R2020b, along with various toolboxes including the Computer Vision Toolbox, ROS Toolbox, and Signal Processing Toolbox. Because the flow depths were derived by subtracting the measured free-surface distance from the measured dry pseudobottom distance, the overall measurement error was minimized. However, actual performance may be affected by the dynamic fast-moving water surface, which could introduce additional uncertainty.

#### 2.4.1. Conversion from Pixel Coordinates to Global 3D Coordinate System

The RGB-D camera captures depth information in pixel-wise coordinates for each recorded scene. While the number of pixels in the *x* and *z* (transverse along the flume width) directions remained constant across recordings, any portion of the fluctuating water surface that is closer to the camera occupied a larger physical area in the FoV and would have a greater pixel density. This poses a challenge for the stepped spillway case where the depth or distance to the water surface changes with respect to time. For this reason, directly averaging depth data over time using pixel-based frames can introduce significant errors. Therefore, to achieve more accurate instantaneous and time-averaged water surface measurements of the camera’s 60 s recording, the pixel data were converted to a 3D point cloud for each recorded frame. This preserved the spatial locations of water surface points and allowed the correct alignment of each frame in 3D space.

To convert the pixel coordinates [x(*i*, *j*), *z*(*i*, *j*), *y*(*i*, *j*)] from the depth image to global 3D coordinates [x(*i*, *j*), *z*(*i*, *j*), *y*(*i*, *j*)], the depth value *y*(*i*, *j*) at each pixel location was used in combination with the camera’s intrinsic parameters: the focal lengths and the principal point coordinates. The global coordinates for a point in space and time of the water surface can be calculated using the following transformation matrix:

(3) $$\left[\begin{array}{c} x\left(i,j\right)\\ z\left(i,j\right) \end{array}\right]=\left[\begin{array}{ccc} \frac{1}{f_{x}} & 0 & -\frac{ppx}{f_{x}}\\ 0 & \frac{1}{f_{z}} & -\frac{ppz}{f_{z}} \end{array}\right]\left[\begin{array}{c} y\left(i,j\right)\times j\\ y\left(i,j\right)\times i\\ y\left(i,j\right) \end{array}\right]$$

where *f_x_* and *f_z_* represent the focal lengths of the camera in the *x* and *z* directions, respectively. Pixel units *ppx* and *ppz* represent the coordinates of the principal point, which is the point of intersection between the image plane and the camera’s optical axis. Equation (3) can be expanded into two separate equations for the *x* and *z* coordinates, which are derived as follows:

(4) $$x\left(i,j\right)=\frac{y\left(i,j\right)\times \left(j-ppx\right)}{f_{x}}$$

(5) $$z\left(i,j\right)=\frac{y\left(i,j\right)\times \left(i-ppz\right)}{f_{z}}$$

where the camera’s intrinsic parameters, including *f_x_*, *f_z_*, *ppx*, and *ppz*, can be extracted from the camera calibration metadata embedded within the “.bag” file of the RGB-D camera recordings. This 3D point cloud conversion had negligible effects on the measurements with the flat wall dataset and may be expected to perform similarly for water surface measurements. Once the 3D point cloud is obtained, it can be further transformed to align with any desired global coordinate system, provided the exact position and orientation of the camera are known. If desired, this pixel-to-3D transformation allows the independent use of multiple cameras and the point clouds could be combined to generate a 3D surface covering a larger area, overcoming the limited FoV constraints associated with a single camera.

#### 2.4.2. Post Processing and Data Filtering

In the initial stage of post-processing, a distance-based rule was applied to exclude physically implausible outliers, discarding depth measurements over 1 m, given that sensors were positioned within 1 m of the pseudobottom of the stepped spillway (clear steps). Following this, a robust outlier filtering technique [49] based on median (MED) and median absolute deviation (MAD) was used to further refine the data, as the time series of flow depth data in a turbulent flow is often known to be accompanied by outliers. Specifically, MED and MAD have a breakdown point of 50% contaminated data vs a 0% breakdown point for mean and standard deviations, with MAD obtained by sorting the absolute value of residuals around MED and selecting the data value corresponding to 50% [49].

Similar to Bung et al. [31], data filtering was conducted across each pixel coordinate (*i*, *j*) to systematically remove outliers from the dataset as follows:

(6) $$y_{filt}\left(t\right)=NaN\;\;\;\;for\left\{\begin{array}{c} y\left(t\right)<y_{min}\\ y\left(t\right)>y_{max} \end{array}\right.$$

(7) $$y_{min}=MED\left(y\left(t\right)\right)-1.4832\times \sqrt{2\;ln\left(N\right)}\times MAD\left(y\left(t\right)\right)$$

(8) $$y_{max}=MED\left(y\left(t\right)\right)+1.4832\times \sqrt{2\;ln\left(N\right)}\times MAD\left(y\left(t\right)\right)$$

where $y_{min}$ and $y_{max}$ represent the lower and upper bounds, respectively; $y\left(t\right)$ is the time series at pixel (*i*, *j*); $MED\left(y\left(t\right)\right)$ is the median and $MAD\left(y\left(t\right)\right)$ is the median absolute deviation of the depth time series. This filtering process was similarly applied to the USS dataset to ensure consistent refinement across data sources.

## 3. Results

### 3.1. Water Surface Observations

**Figure 5:**
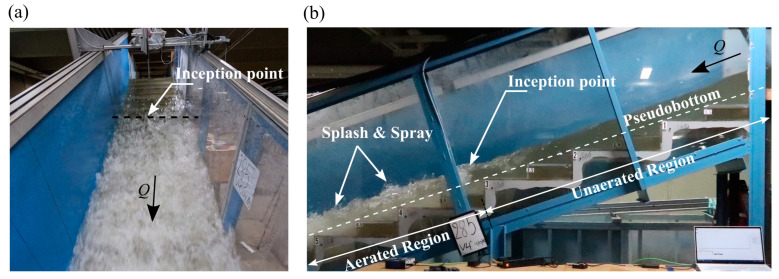
Flow features and inception point location for *q* = 0.285 m^2^/s. (**a**) Top view; (**b**) side view.

As part of quantifying what the RGB-D camera is measuring, the following description of the water surface in the stepped spillway is provided. The flow exhibited the typical skimming flow pattern over a stepped spillway [13,50] with the expected upstream unaerated clear water region and the downstream aerated white-water region. The inception point, which refers to the transition zone between these two distinct flow regions [51], was located near step edge 3.5 (*x* = 2.214 m) and was characterized by rapid aeration and subsequent flow bulking (see Figure 5a,b).

**Figure 6:**
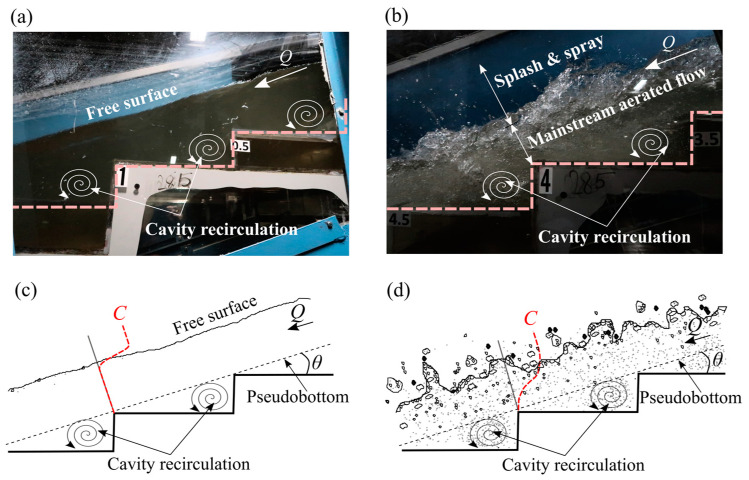
Detailed view of the skimming flow over the stepped chute along with schematic representation of the clear water (**a**,**c**) and aerated region (**b**,**d**).

Flow entering the stepped spillway was transparent with a very smooth water surface. The inception point included roughening of the water surface, self-aeration, and entrainment of large quantities of air into the step cavities. Downstream of the inception point water surface undulations increased due to the development of water surface perturbations [52] that entrapped some air near the free surface. In addition, considerable splash spray was observed in the aerated region where water droplets of varying sizes were ejected. The surface in this region was highly distorted and fluctuating, lacking a well-defined free surface (Figure 6b,d) and exhibiting dynamic behavior throughout. In contrast, the upstream unaerated section exhibited a distinct continuous free surface without distortions and free surface undulations (Figure 6a,c). Note that, in Figure 6c,d, typical air concentration (*C*) profiles as obtained with the PDCP are included for reference.

In the aerated region downstream of the inception point, a distinct water surface is lacking and traditionally this is defined with PDCP measurements with the flow depth at 90% air concentration (*y*_90_) serving as a standard reference [50].

**Figure 7:**
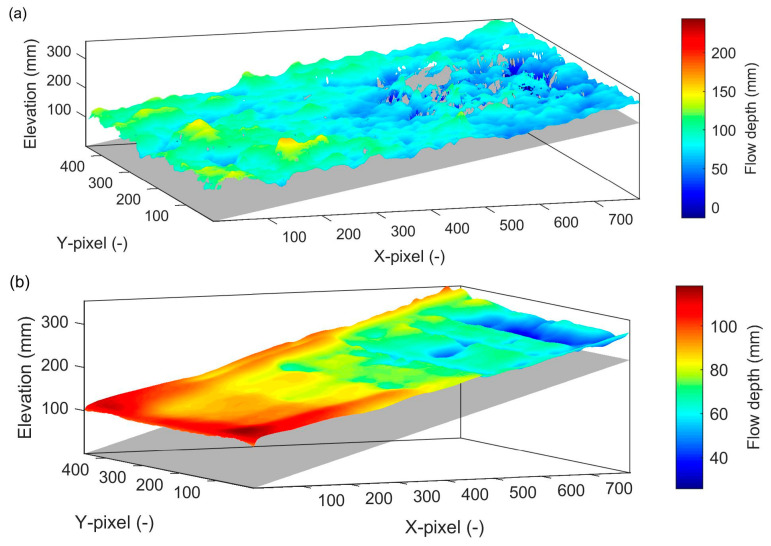
Three-dimensional water surface topography captured by the RGB-D camera in pixel coordinates. (**a**) Instantaneous water surface; (**b**) time-averaged mean water surface.

Figure 7a shows the dynamic water surface captured by the RGB-D camera for an instantaneous water surface in pixel coordinates. This aligns with the known requirements of the RGB-D camera for depth estimation, which relies on well-defined features for stereo matching. The RGB-D camera is known to face challenges for shiny and transparent objects or underexposed scenes [53]. Although water surface fluctuations were detected, the camera appears to not have sufficient resolution or frame rate to detect small water droplets or potentially some may appear transparent. A region of no-data points was also observed in the unaerated flow region due to a lack of detectable water surface features. Figure 7b shows a 3D surface from the RGB-D camera averaged over *T* = 60 s. The time-averaged 3D surface was free from no-data points unlike the instantaneous topography. In the aerated region, the camera produced realistic flow depths as the camera was able to capture the turbulent water features, facilitated further by an opaque water surface whereas the unaerated region showed unrealistically smaller flow depths due to transparency.

### 3.2. Water Surface Profiles

**Figure 8:**
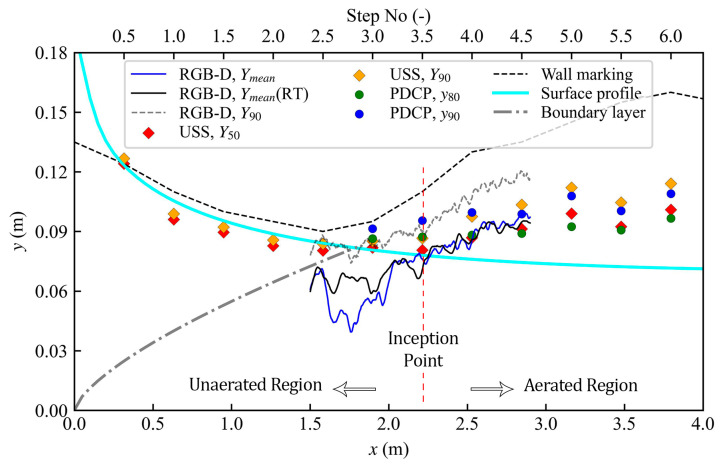
Water surface profile comparison using different sensors and techniques. Theoretical profile for unaerated flow based on Felder and Chanson [54] and boundary layer thickness from Zhang and Chanson [55].

Wall markings of the visually estimated water surface profile at the viewing window are provided in Figure 8. Also included in Figure 8 are water surface profiles via measurements using the RGB-D sensor (a continuous water surface profile was obtained simultaneously from the RGB-D camera recordings along the centerline and right transect (see Figure 3) of the flume) and the discrete USS measurements at step edges. Also shown in Figure 8 are the PDCP data and a theoretical profile of the water surface assuming unaerated flow [54]. Finally, the boundary layer thickness is also presented, based on Zhang and Chanson [55], for stepped spillways with a broad crested weir given as follows:

(9) $$\frac{\delta }{x}=0.15{\left(\frac{x}{k_{s}}\right)}^{-0.311}$$

where δ is the boundary layer thickness, x is the streamwise distance from the broad crested weir, and $k_{s}=hcos\theta$ is the macro-roughness due to the steps. The boundary layer originates at the broad crested weir and grows in the downstream direction, intersecting the free surface some distance down the chute.

Upstream of the inception point (*x* < 2.214 m; see Figure 8), the RGB-D camera measurements produced mean flow depths (*y_mean_*) lower than the visually observed free-surface and the measurements of USS (*Y*_50_). This discrepancy likely stems from the limitations of the camera in the absence of adequate surface texture or opacity from aeration; water transparency allows the steps below to remain visible in the FoV. Due to the transparency, the stereo-matching algorithm, which relies on clear, distinguishable features in the scene, often “locks onto” the underlying steps instead of the true free surface. However, the PDCP standard of considering *y*_90_ as a representative water surface [11,13] suggests that the camera intermittently captures the free surface in this region, which may be linked to the sporadic surface perturbations creating some texture on the flow surface facilitating occasional detection. Immediately upstream of the inception point, slight air pockets within the surface layers are occasionally trapped, potentially offering enough contrast for intermittent surface detection by the camera. Also, it was seen that the transparency was dynamic as the camera could occasionally detect the water surface in the clear water region as supported by *Y*_90_ of the camera. Nonetheless, the RGB-D *Y_mean_* values were consistently lower in the unaerated region, which highlights the limitation of the camera for detecting transparent objects. For instance, at Step 3 (*x* = 1.897 m), the percent difference (ε) between the PDCP (0.091 m) and RGB-D (0.055 m) reaches 39.6%, and the difference with USS (0.082 m) is about 32.5%. Likewise, at *x* = 2.214 m, the PDCP–RGBD difference remains relatively high (20.3%), though the USS–RGBD difference is smaller (5.6%). The measurement capabilities of the camera in the transparent region could be improved by increasing the surface contrast using some floating tracers or making the flow opaque using color dyes.

Downstream of the inception point (*x* > 2.214 m), where detectable surface features were prevalent and air entrainment increased the water’s opacity, the RGB-D sensor showed better agreement with the USS and the flow depths remained close to *y*_80_ to *y*_90_ from the PDCP. For instance, at *x* = 2.530 m, the differences between RGB-D (0.085 m) and PDCP (0.100 m), or USS (0.087 m), are 14.4% and 1.9%, respectively. Farther downstream, at *x* = 2.846 m, the flow depths from PDCP (0.09876 m) and USS (0.091 m) remained close to the RGB-D (0.099 m), resulting in a PDCP–RGBD difference of about −0.3% and a USS–RGBD difference of −8.6 %. Bung et al. [31] observed slightly lower flow depth measurements for USS than the RGB-D camera in a highly aerated hydraulic jump. For a stepped spillway case, Bung [22] reported that the *Y*_50_ measurements with the USS were closer to the *y*_80_ of the conductivity probe, which is the characteristic depth at *C =* 0.8, a trend also observed in this study. This shift may be explained by the USS emitting high-frequency sound waves that may penetrate slightly the observed water surface [22,56]. It is notable that the camera surface profiles for the centerline and the right transect (RT) showed good agreement, although some differences appeared upstream of the inception point where the measurements could be affected due to transparency and varying levels of reflectivity of the water surface. In general, the flow depths are uniform within a cross-section, and the close agreement between the surface profiles along the two transects highlights the 3D capabilities of the RGB-D camera.

**Figure 9:**
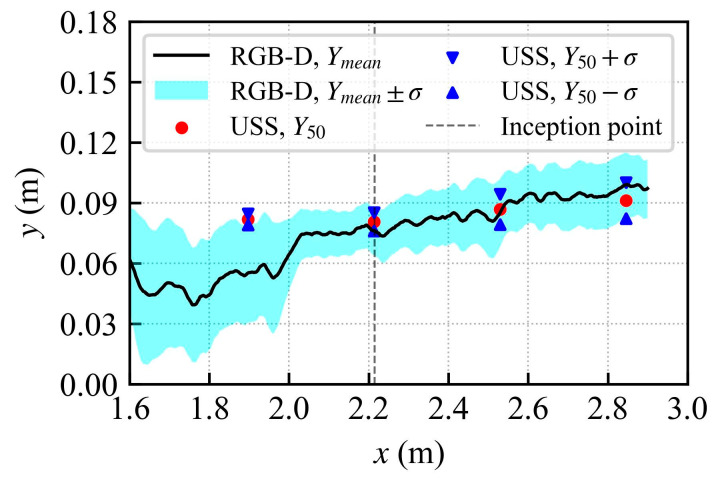
Standard deviations of the measured water surface along the centerline of the flume.

Figure 9 provides the standard deviations of the water surface profile in the streamwise direction. The water surface fluctuations rapidly increased downstream of the inception point as the air was entrained, followed by flow bulking and splash/spray. The USS measurements exhibited smaller deviations of the water surface near the inception region and upstream; however, it increased in the downstream direction. Similar is the case with the camera measurements; however, further upstream of the inception point, the camera performs poorly, occasionally picking up the water surface but, in most instances, it captures flow depth further below the water surface; hence, larger standard deviations occur opposite from the actual behavior of the flow.

### 3.3. Water Surface Fluctuations

**Figure 10:**
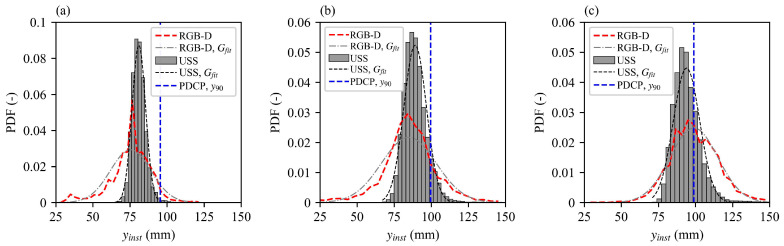
PDF distributions of the time series of flow depths measured with USS and RGB-D camera. (**a**) Step 3.5, (**b**) Step 4, and (**c**) Step 4.5. Note that *G_fit_* stands for the Gaussian fit for the datasets.

The probability density function (PDF) distributions of flow depths from non-intrusive techniques (USS and RGB-D) together with *y*_90_ from the PDCP at the step edges are presented in Figure 10. In general, both the USS and RGB-D measurements exhibited distributions that approximate a Gaussian shape centered around their respective man flow depths, suggesting that the measured water surface oscillates predictably within this pattern. Notably, the camera data reveal a wider range of flow depths compared to the USS measurements, with both minimum and maximum depths extending beyond the limits observed by the USS. As the flow advances downstream, the extent of surface fluctuations broadens, driven by escalating water surface turbulence as self-aeration intensifies near the inception point. This progression underscores the effect of water surface aeration and subsequent turbulence on surface variability, accentuating the spatial dynamics of the water surface in the streamwise direction. The performance of the camera compared to the USS in this study is different from the findings of Bung et al. [31] for a classical hydraulic jump case, where the camera was found to exhibit a narrower band of fluctuations than the USS. This indicates that the relative performance of stereoscopic vision based optical sensing methods may vary depending on the specific flow configuration and aeration levels. For instance, in Figure 10a, the smallest camera-recorded depth (~25mm) is smaller than the actual minimum flow depth observed experimentally. However, in the more highly aerated downstream region where the flow becomes distinctly “milky white”, the camera’s minimum depth readings increase, reflecting improved accuracy under higher aeration. Additionally, the mean depths provided by the camera align more closely with the PDCP *y*_90_ depths, as also seen in Figure 8, further illustrating the camera’s enhanced reliability in fully aerated conditions.

### 3.4. Air Concentrations and Interface Frequencies

To further evaluate the capabilities of this RGB-D sensor, *C* profiles and interface count rates (*Fs*) were computed from the instantaneous camera depth dataset. Firstly, the continuously recorded flow depth time series was converted into a time series of instantaneous *C* at specified elevations above the pseudobottom using the criteria similar to Kramer and Bung [16] and Cui et al. [17] as follows:

(10) $$C\left(t\right)=\left\{\begin{array}{c} 1\;for\;y\left(t\right)<y_{ref}\\ 0\;for\;y\left(t\right)\geq \;y_{ref} \end{array}\right.$$

where *C*(*t*) is the instantaneous air concentration, *y*(*t*) is the instantaneous flow depth, and *y_ref_* is the reference flow depth for which *C* is being estimated. Subsequently, *C*(*t*) at each reference elevation was used to compute the time-averaged air concentrations *C* as follows:

(11) $$C\left(y\right)=\frac{1}{T}\int_{t=0}^{t=T}C(t)\;dt$$

*C* measurements by the non-intrusive measurement techniques may be understood based on Kramer and Valero’s [57] two-layer air transport framework, which models aerated flow as a combination of a turbulent boundary layer (TBL) and a turbulent wavy layer (TWL) where the latter corresponds to the water surface region characterized by surface fluctuations and waves. The non-intrusive techniques in this study are expected to be handicapped for air concentration measurements in the TBL region. However, in principle, they should capture the water surface in the TWL providing measurements for the entrapped air concentrations. Meanwhile, PDCP measures both the entrapped and entrained air within the TWL.

**Figure 11:**
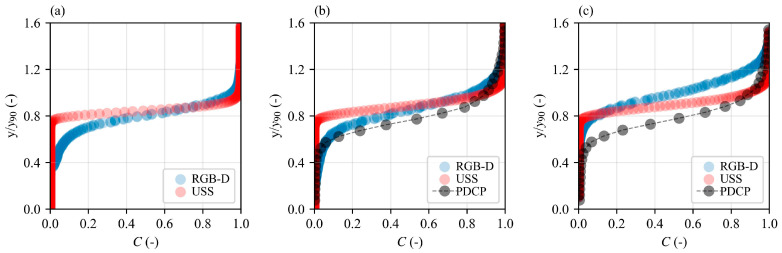
Comparison of time-averaged air concentration distribution along normalized depths for USS, RGB-D camera and PDCP. Here, *y_90_* is the flow depth at 90% air concentration based on PDCP measurements; (**a**) Step 3.5, (**b**) Step 4, and (**c**) Step 4.5.

Figure 11 shows the distribution of *C* profiles at step edges downstream of the inception point. The profiles exhibited characteristic S-shaped curves across normalized flow depths (*y*/*y*_90_), with notable distinctions between measurement approaches. Variations between RGB-D and USS relative to PDCP measurements are anticipated, as PDCP captures both entrained and entrapped air, while the RGB-D and USS methods, being non-intrusive, reflect only entrapped air concentrations within the TWL of the flow.

Again, the results are different from the previous results of Kramer and Bung [16] for the USS measurements. This might be due to the placement of the USS at ≈0.96 m which increased the detection zone of the USS, resulting in measurements outside the point of interest. Positioning the sensor closer to the water surface could mitigate this limitation. Nevertheless, the camera data show promising results as they consider a single pixel location and closely adhere to the concept of the two-state superposition principle of Kramer and Valero [57].

**Figure 12:**
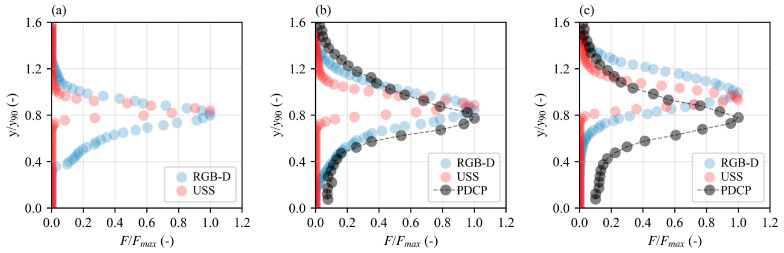
Air–water interface frequency distribution measured with RGB-D camera, USS, and PDCP for *q* = 0.285 m^2^/s. (**a**) Step 3.5, (**b**) Step 4, and (**c**) Step 4.5.

Furthermore, the results from the implementation of Equation (10), which yielded a time series of binary states representing transitions between air and water phases at each depth level, were further analyzed to estimate interface frequencies. Interface frequencies were then calculated by counting transitions from 0 (air) to 1 (water), with each transition signifying a crossing of the water surface. These transition counts, recorded at each depth level, provided a detailed profile of interface frequencies as a function of normalized depth above the pseudobottom (Figure 12).

Although the interface was detected for a wider range of depths for RGB-D, the maximum interface frequency (*F*/*F_max_*) occurred at similar depths for both the non-intrusive techniques. Understandably, *F*/*F_max_* occurred at smaller flow depths for the PDCP and further below for increasing air concentration from Step 4 to Step 4.5. PDCP detects the large numbers of air bubbles entrained in the mainstream flow with the bubble counts increasing from close to zero at the pseudobottom and increasing towards a maximum value in the intermediate flow region 0.3 < *C* < 0.7 [50] while decreasing back to zero at the water surface. The maximum interface count rates for the three techniques were in the order ${\left(F_{max}\right)}_{PDCP}>{\left(F_{max}\right)}_{RGBD}>{\left(F_{max}\right)}_{USS}$. While there are quantitative differences among the flow techniques, all of them provide some account of the dynamic fluctuations of the water surface.

## 4. Conclusions

This study presents the application of an Intel^®^ RealSense™ D455 RGB-D camera for measuring the water surface of a highly turbulent, aerated flow over a stepped spillway, offering insights into its suitability as a non-intrusive alternative to conventional methods. The camera’s performance was shown to be sensitive to ambient lighting conditions, particularly when surfaces were underexposed or exhibited strong reflectivity. Thus, careful control of lighting sources and positioning becomes essential for reliable depth acquisition. The water surface measurements were conducted using a custom preset at a resolution of 848×480 for 60 s at a frame rate of 90 Hz. The RGB-D camera was able to scan the complex water surface and capture its topography in detail. However, varying levels of performance in the unaerated and fully aerated regions of the flow were identified. In the transparent unaerated region, the camera performed poorly and was not able to accurately capture the water surface, whereas fully aerated flow provided enough texture and opacity for water surface detection by the camera.

The comparison with the intrusive phase detection technique provided direct validations to the characteristic levels detected by the RGB-D camera for the first time. In the fully aerated region downstream of the inception point, the mean flow depth measured by the RGB-D camera was close to the flow depths at 80–90% air concentration levels. The accuracy of the RGB-D camera was affected in the unaerated transparent regions, with the detected flow depths consistently lower than the USS and PDCP measurements. In the fully aerated region (step edge 4.5), the flow depths from the camera were comparatively greater than the USS measurements and were in the order PDCP > RGB-D > USS, with the camera measurements within 10% of the USS measurements. The RGB-D camera was also able to capture the temporal fluctuations of the water surface, providing insights into interfacial frequencies and entrapped air concentration distribution. Although the limitation of the camera in the transparent region was evident, it performed well towards the aerated region, and the question “what does the camera capture?” in an aerated scene was answered. Overall, this study suggests that stereoscopic vision-based photogrammetry, as employed by RGB-D cameras, could serve as a promising tool for spatio-temporal measurement of flow depths in highly aerated flows, expanding the scope for non-intrusive measurement techniques in turbulent water environments. This can be particularly useful when a simultaneous repeated measurement at multiple locations is required. However, to use this camera technique, the flow surface should have distinct features for detection. To further generalize the measurement capabilities of this camera technique, more testing is recommended under varying turbulence conditions, different camera models, and free surface topography. Integration with machine learning-based post-processing techniques could further refine surface detection by these cameras. Such focused studies would help establish robust guidelines and expanded use cases for RGB-D technology in hydraulic research and engineering practice.

## Figures and Tables

**Table 1:** Relevant studies featuring non-intrusive methods to analyze turbulent water surfaces.

Reference	Non-Intrusive Methods Used	Application Area
Kramer and Bung (2024) [16]	Laser point sensor	Hydraulic jump and stepped spillway
Cui et al. (2022) [17]	USS	Grass lined spillway surface
Bung (2013) [22]	USS/ High speed camera	Stepped spillway/Smooth invert chute
Nina et al. (2022) [27]	High speed camera	Stepped spillway
Pacloccic et al. (2020) [28]	High speed camera/LiDAR	Channel confluence
Kramer and Felder (2021) [29]	High speed camera	Stepped spillway
Montano et al. (2018) [26]	LiDAR	Hydraulic jump
Pleterski et al. (2023) [30]	RGB camera array	Supercritical junction flow
Steinke and Bung (2024) [25]	USS/RGB-D Camera/4D PTV	Undular hydraulic jump
Bung et al. (2021) [31]	RGB-D camera	Hydraulic jump

**Table 2:** Features and specifications of Intel^®^ RealSenseTM D455 depth camera. Note: *H_FoV_* = horizontal field of view, *V_FoV_* = vertical field of view, and *D_FoV_* = diagonal field of view.

Features	Specifications
Nominal Dimensions (width × height × depth)	124 mm × 26 mm × 29 mm
Depth Field of View	*H_FoV_*: 87° ± 3°, *V_FoV_*: 58° ± 1°, *D_FoV_*: 95° ± 3°
Depth Frame Rate	90 fps (maximum)
RGB Field of View	*H_FoV_*: 90° ± 1°, *V_FoV_*: 65° ± 1°, *D_FoV_*: 98° ± 3°
RGB Frame Rate	60 fps (maximum)
Infrared Projector Field of View	*H_FoV_*: 90°, *V_FoV_*: 63°, *D_FoV_*: 99°
Ideal Range	0.6 m to 6 m
RGB Resolution (maximum)	1920 × 1080
Depth Resolution (maximum)	1280 × 720
Baseline Distance	95 mm
Depth Technology	Stereoscopic
RGB sensor Technology	Global Shutter
Depth Accuracy	<2% at 4 m

**Table 3:** Summary of camera settings and parameter ranges.

Parameter	Range/Value	Description/Notes
Visual presets	Custom, Default, Hand, High accuracy, High density, Medium density	A custom preset was selected based on better fill rates and resulting depth map
Sampling frequency (*f_RGB_*)	30–90 Hz	Higher frame rates capture rapid free-surface fluctuations. Adopted: 90 Hz
Image resolution	Multiple, e.g., 848 × 100, 848 × 480, 1280 × 720, 1280 × 800, etc.	Selected 848 × 480 pixels to balance field of view and spatial resolution.
Laser power	250 mW (range: 0–360 mW)	Higher power improved depth detection in aerated regions; set per Bung et al. [31] and Carfagni et al. [41]
Depth unit	1 mm	Defines internal quantization scale of depth
Depth exposure	33,000 μs (range: 10,000–40,000 μs)	Adjusted to optimize depth sensing for lighting conditions used in this study
Image gain	Minimum set to 16 (range: 0–64)	Helps balance brightness in challenging lighting or high exposure scenarios

**Table 4:** Specifications and locations of the light sources used to illuminate the flume for free-surface measurements. The table lists each light’s approximate placement relative to the flume geometry, the type and shape of the fixture, its power rating, and the emitted light color.

S. No.	Location	Type	Shape	Rating	Light Color
L1	~0.8 m above weir crest	LED	1-m Linear (2 rows)	500 W	White
L2	~1 m above step edge 3.5	LED	10″ Ring	10 W	White
L3	~2.4 m above step edge 6	LED	1-m Linear (2 rows)	500 W	White
L4	Underneath steps	LED	1 m Linear (2 rows)	500 W	White

## Data Availability

Upon reasonable request, either some or all of the data, model, or codes supporting the findings of this study can be provided by the corresponding author.
